# Agreement between cystatin-C and creatinine based eGFR estimates after a 12-month exercise intervention in patients with chronic kidney disease

**DOI:** 10.1186/s12882-018-1146-4

**Published:** 2018-12-18

**Authors:** Kassia S. Beetham, Erin J. Howden, Nicole M. Isbel, Jeff S. Coombes

**Affiliations:** 1 School of Human Movement and Nutrition Sciences, The University of Queensland, Brisbane, Queensland Australia; 2 School of Medicine, The University of Queensland, Brisbane, Queensland Australia; 3 School of Exercise Science, Australian Catholic University, Brisbane, Queensland Australia; 4 Baker Heart and Diabetes Institute, Melbourne, Victoria Australia; 5 Department of Nephrology, Princess Alexandra Hospital, Brisbane, Queensland Australia

**Keywords:** Chronic kidney disease, Renal, Kidney function

## Abstract

**Background:**

Estimation of GFR (eGFR) using formulae based on serum creatinine concentrations are commonly used to assess kidney function. Physical exercise can increase creatinine turnover and lean mass; therefore, this method may not be suitable for use in exercising individuals. Cystatin-C based eGFR formulae may be a more accurate measure of kidney function when examining the impact of exercise on kidney function. The aim of this study was to assess the agreement of four creatinine and cystatin-C based estimates of GFR before and after a 12-month exercise intervention.

**Methods:**

One hundred forty-two participants with stage 3–4 chronic kidney disease (CKD) (eGFR 25–60 mL/min/1.73 m^2^) were included. Subjects were randomised to either a Control group (standard nephrological care [*n* = 68]) or a Lifestyle Intervention group (12 months of primarily aerobic based exercise training [*n* = 74]). Four eGFR formulae were compared at baseline and after 12 months: 1) MDRDcr, 2) CKD-EPIcr, 3) CKD-EPIcys and 4) CKD-EPIcr-cys.

**Results:**

Control participants were aged 63.5[9.4] years, 60.3% were male, 42.2% had diabetes, and had an eGFR of 40.5 ± 8.9 ml/min/1.73m^2^. Lifestyle Intervention participants were aged 60.5[14.2] years, 59.5% were male, 43.8% had diabetes, and had an eGFR of 38.9 ± 8.5 ml/min/1.73m^2^. There were no significant baseline differences between the two groups. Lean mass (*r* = 0.319, *p* < 0.01) and grip strength (*r* = 0.391, *p* < 0.001) were associated with serum creatinine at baseline. However, there were no significant correlations between cystatin-C and the same measures. The Lifestyle Intervention resulted in significant improvements in exercise capacity (+ 1.9 ± 1.8 METs, *p* < 0.001). There were no changes in lean mass in both Control and Lifestyle Intervention groups during the 12 months. CKD-EPIcys was considerably lower in both groups at both baseline and 12 months than CKD-EPIcr (Control = − 10.5 ± 9.1 and − 13.1 ± 11.8, and Lifestyle Intervention = − 7.9 ± 8.6 and − 8.4 ± 12.3 ml/min/1.73 m^2^), CKD-EPIcr-cys (Control = − 3.6 ± 3.7 and − 4.5 ± 4.5, and Lifestyle Intervention = − 3.6 ± 3.7 and − 2.5 ± 5.5 ml/min/1.73 m^2^) and MDRDcr (Control = − 9.3 ± 8.4 and − 12.0 ± 10.7, Lifestyle Intervention = − 6.4 ± 8.4 and − 6.9 ± 11.2 ml/min/1.73 m^2^).

**Conclusions:**

In CKD patients participating in a primarily aerobic based exercise training, without improvements in lean mass, cystatin-C and creatinine based eGFR provided similar estimates of kidney function at both baseline and after 12 months of exercise training.

**Trial registration:**

The trial was registered at www.anzctr.org.au (Registration Number ANZCTR12608000337370) on the 17/07/2008 (retrospectively registered).

**Electronic supplementary material:**

The online version of this article (10.1186/s12882-018-1146-4) contains supplementary material, which is available to authorized users.

## Background

The most common approach used to assess kidney function is by estimation of glomerular filtration rate (eGFR) based on serum creatinine concentrations, as it can be calculated routinely from standard tests and is inexpensive. Physical exercise can cause an increase in serum creatinine; [1] therefore this method may not be suitable for use in exercising individuals. Alternatively, cystatin-C based eGFR is suggested to be an improved method for eGFR measurement and may provide a more precise measure of kidney function [2]. The agreement between cystatin-C and creatinine based eGFR before and after an exercise intervention in chronic kidney disease (CKD) patients is yet to be studied.

The current reference method for measuring kidney function is to quantify the clearance of an exogenous marker, such as inulin, to determine GFR [3]. However, this measure is rarely used clinically due to the test complexity and cost. Instead, indirect assessment of endogenous markers to provide an estimation of GFR is commonly used for routine clinical measurements via different formulas [4]. These formulae use either a measure of creatinine or cystatin-C in serum or urine, along with patient characteristics; age, sex and race to estimate filtration rates. The four most frequently used eGFR equations are: 1) modification of diet in renal disease-creatinine (MDRDcr) [5], 2) chronic kidney disease-epidemiology collaboration (CKD-EPI)cr [6], CKD-EPIcystatin-C(cys) [7] and CKD-EPIcr-cys [7].

Creatinine based eGFR measures are commonly used as a ‘first test’ and for routine clinical assessment of kidney function [7, 8]. Creatine phosphate is taken up by muscle after it is released into the circulatory system following synthesis in the liver. Creatinine is formed as the by-product of muscle creatine breakdown during muscle contraction [9] and therefore can be directly influenced by muscle mass [10, 11]. This is an important limitation for estimation of GFR in a population such as CKD who typically have reduced muscle mass [11]. Furthermore, intense exercise may cause a breakdown of muscle leading to an increase in serum creatinine levels, [1] potentially making creatinine-based eGFR measures inaccurate in exercising individuals. Cystatin-C is a low molecular weight cystine protease inhibitor that is produced at a constant rate by all nucleated cells and is not influenced by muscle mass [12]. The use of cystatin-C to calculate eGFR is suggested by some as an alternative measure of eGFR due to its increased association with risk of death and progression to end-stage renal disease [13].

Exercise training is important for patients with CKD as it has been shown to improve physical function and cardiovascular risk factors in a number of studies [14–16]. Therefore, it is important to identify whether there is a difference in the agreement between cystatin-C and creatinine based eGFR measures after exercise-induced adaptations occur. It is also important to assess the impact of exercise training on kidney function – such as by slowing the rate of decline or the potential for harm by volume depletion.

The aim of this study was to assess the agreement between cystatin-C and creatinine based eGFR estimates following a primarily aerobic exercise intervention. It was hypothesised that there would be poor agreement between these measures after 12 months of exercise training due to the impact of exercise on muscle breakdown and thereby serum creatinine.

## Methods

**Fig. 1:**
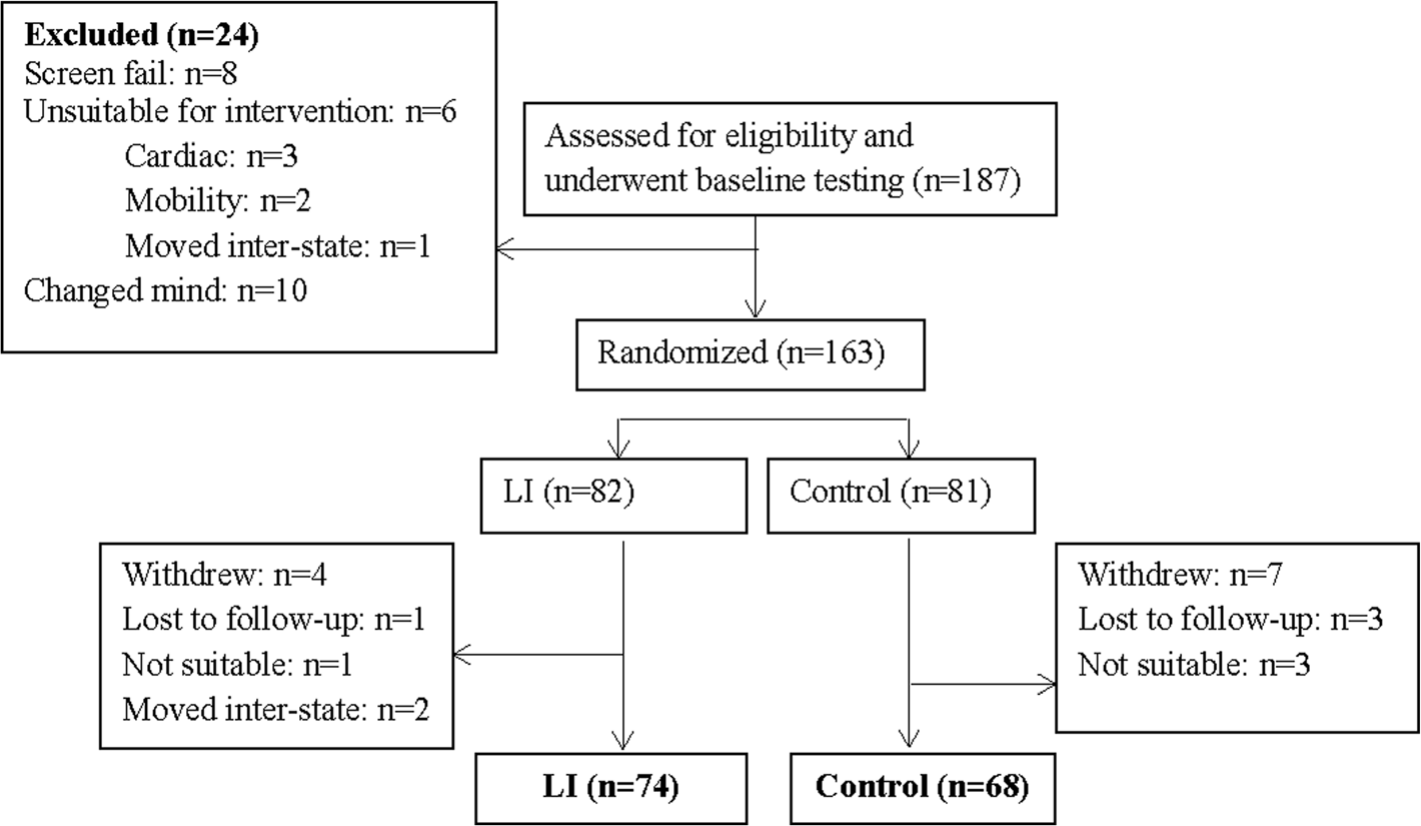
Consort diagram

The data from this study is a 12-month analysis from the ‘LANDMARK III’ study (**L**ongitudinal **A**ssessment of **N**umerous **D**iscrete **M**odifications of **A**therosclerotic **R**isk **K**idney disease), a randomised control trial looking at the effects of a 3-year multi-disciplinary lifestyle intervention in patients with CKD. This study included 142 subjects with stage 3–4 CKD (MDRDcr-_175_ eGFR 25–60 mL/min/1.73m^2^) (Fig. 1). Inclusion criteria: aged 18 to 75 years and at least one of the following risk factors – blood pressure or lipids not at target; overweight (body mass index [BMI] > 25 kg/m^2^); and poor diabetic control (haemoglobin A1c > 7%). Exclusion criteria were: intervention for, or, symptomatic coronary artery disease (within 3 months), current heart failure (New York Heart Association class III and IV) or significant valvular heart disease, pregnant or planning to become pregnant and life expectancy or anticipated time to dialysis or organ transplant < 6 months. Participants were asked to refrain from any exercise on the morning of the test.

### Exercise program

Intervention participants were treated by a multidisciplinary team consisting of Exercise Physiologists, Nurse Practitioners, Dieticians, Diabetic Educator and Nephrologists. Participants trained for 8 weeks, a minimum of twice per week with an Exercise Physiologist in a supervised gym-based setting. The gym-based training incorporated a combination of aerobic and resistance exercises. After the 8 weeks of gym-based training, patients completed a home program for 10 months with regular gym refresher sessions, and regular follow-up via telephone and email. The home program consisted of resistance exercises based on a provided thera-band and swiss ball, with a large emphasis on aerobic training. A more detailed description of the lifestyle intervention is outlined in Howden et al. (2013) [17].

### Creatinine and cystatin-C measurements

Serum vacutainers (BD vacutainers, NJ, USA) were used to collect 10 mL venous blood samples following an overnight fast. Creatinine was measured by the Jaffe method on a Beckman DxC800 general chemistry analyser (Beckman Coulter Diagnostics, Brea, CA, USA). A particle-enhanced immunoturbidimetric assay (Cystatin-C Tina-quant Roche/Hitachi- Roche Diagnostics GmbH. Mannheim Germany) on a COBAS Mira clinical autoanalyser (Roche Diagnostica Switzerland), was used to perform serum cystatin-C measurements according to the manufacturer’s instructions. Control and calibration materials were provided by Roche Diagnostics. A 2-reagent assay system was used, in where reagent 1 is a buffer and reagent 2 is a suspension of latex particles coated with rabbit anti-cystatin-C specific polyclonal antibodies. The sample is mixed with reagent 1 in a cuvette and incubated for 3 min, after which reagent 2 is added. After 7.5 min from the start of the cohesion reaction, of wavelength 550 nm, the absorbance difference was measured. All cystatin-C measures were performed in duplicate and the average was taken. If there was a difference of greater than 10% between 2 measures, a 3rd measure was taken and if the 3 values were similar, the average was taken, or if an outlier of the 3 was detected it was deleted. Our intra-assay coefficient of variation based on duplicate measures was 3.8%.

### eGFR measurements

Four measures of renal function were assessed by eGFR; MDRDcr_– 175_ study eq. (2006), [18] CKD-EPIcr eq. (2009), [6] CKD-EPIcys eq. (2012) [7] and CKD-EPIcr-cys eq. (2012) [7]. Table 1 outlines the calculations used for each formula. All eGFR’s are made relative to a surface area of mL/min/1.73m^2^.

**Table 1:** eGFR equations

Name	Sex	Cr	Cys	Equation
MDRD (2006) [18]				175xcr^-1.154^xage^-0.203^(× 0.742,if female)(× 1.212,if black)
CKD-EPIcr (2009) [6]	F	≤0.7		144x(cr/0.7)^-0.329^ × 0.993^age^(× 1.159,if black)
		>0.7		144x(cr/0.7)^-1.209^ × 0.993^age^(× 1.159,if black)
	M	≤0.9		141x(cr/0.9)^-0.411^ × 0.993^age^(×1.159,if black)
		>0.9		141x(cr/0.9)^-1.209^ × 0.993^age^(×1.159,if black)
CKD-EPIcys (2012) [7]	F		≤0.8	133x(cys/0.8^)-0.499^ × 0.996^age^x0.932
			>0.8	133x(cys/0.8^)-1.328^ × 0.996^age^x0.932
	M		≤0.8	133x(cys/0.8^)-0.499^ × 0.996^age^
			>0.8	133x(cys/0.8^)-1.328^ × 0.996^age^
CKD-EPIcr-cys (2012) [7]				130x(cr/0.7)-0.248x(cys/0.8)-0.375 × 0.995age(×1.08,if black)

*MDRD* modification of diet in renal disease, *CKD-EPI* chronic kidney disease epidemiology collaboration, *cr* creatinine, *cys* cystatin-C, *F* female, *M* male

### Fitness measures

A hand grip dynamometer was used to assess grip strength (Jamar 5030 J1, Illinois, United States). The maximum grip strength was recorded from the highest of three readings of either hand. An improvement in grip strength was determined by an increase from baseline to 12 months. Peak oxygen uptake (VO_2_peak) measured by expired air analysis was used to assess cardiorespiratory fitness (Vmax29c, SensorMedics, CA, USA), using the peak 20 s average of the final minute during a maximal treadmill test. The test protocol used was either Bruce, Balke or Naughton protocols, based on participants responses to the Duke Activity Status Index, and the same test was performed pre and post intervention [19]. Exercise capacity was determined from the treadmill test as estimated metabolic equivalent tasks (METs) (GE Case V6.51, Wisconsin, USA) and from a six-minute walk test. Muscular power was measured using the Get up and Go test, from a 3-m timed lap from a non-armed chair. The best time in seconds from three successive trials was used as the final time.

### Physical activity

The self- reported Active Australia (AA) questionnaire was used to assess average weekly physical activity levels from the preceding six months. [20] Questions from the AA used in the data analysis are; average weekly time, in the past six months, for time spent walking (for at least 10 min without stopping), time spent doing moderate intensity activity and time spent doing vigorous activity. The questions were asked by an Accredited Exercise Physiologist to avoid any misunderstanding by the subject and clarity was checked on each question. Participants were required to provide examples of the activity that they reported in each of the three categories (walking, moderate and/or vigorous) in order to limit over-representation of an activity. Standardized examples for each category (eg. moderate = gentle swimming/social tennis/golf; vigorous = jogging/cycling/aerobics/competitive tennis) were provided to the participant as suggested on the AA questionnaire.

The metabolic equivalent of a task provides an estimate of energy cost to a physical activity [21]. Therefore, an activity of higher intensity would have a higher MET score. According to the International Physical Activity Questionnaire, MET intensities were calculated by time in each intensity multiplied by either 8 (vigorous), 4 (moderate) and 3.3 (walking), respectively (www.ipaq.ki.se). Total MET hours are calculated by the addition of vigorous MET hours, moderate MET hours and walking MET hours. An improvement in total MET hours is determined by an increase from baseline to 12 months.

### Lean mass

Dual energy x-ray absorptiometry (DEXA), using whole body composition analysis was used to assess lean mass (Hologic QDR 4500A Version 12.6, Massachusetts, USA). To assess skeletal muscle separate to lean tissue, [22] trunk lean mass was removed from the lean mass total. As such, lean mass was estimated from analysis from the appendicular skeleton and the average of the four limbs was calculated. DEXA was performed on a representative sub-set of patients due to limited machine availability (*n* = 73). The DEXA was performed prior to the exercise stress test on the day of testing.

### Statistics

Mean ± standard deviation (SD) was used to describe normally distributed baseline characteristics, with percentages used to describe frequencies for categorical variables. Median [IQR] was used to describe not normally distributed variables and variables transformed by the natural logarithm. Pearson’s correlation was used to assess association for normally distributed and log transformed variables. Comparison between groups was assessed by an independent t-test of the delta from baseline to 12 months. Mann-Whitney U test was performed on not-normally distributed variables. Differences between groups for categorical variables were analysed using Pearson’s Chi Square test. Within group differences were assessed by paired t-tests for normally distributed and log transformed variables. Wilcoxon-Sign rank test was used for not normally distributed variables. A multiple linear regression was used to identify independent correlates with change in eGFR measures, using the enter method. Bland-Altman analysis was used to assess agreement between eGFR measurements using GraphPad Prism 7. All other statistical analyses were performed on IBM SPSS Statistics 22. With many repeated variables in a large sample size, a small amount of missing data for variables were inevitable. Participants were included in the analysis (ie. *n* = 142) if they had either cystatin-C or creatinine measures (and therefore their subsequent eGFR measures) at both baseline and 12 months. Statistical significance was set at *p* < 0.05.

## Results

### Patient characteristics

There were no significant differences between groups for any of the baseline characteristics or medication use (Table 2). Patients were on average 62 years of age and generally obese with an average BMI of 32 kg/m^2^. Mean VO_2_peak for the group was considered very poor, according to the American College of Sports Medicine (2010) normative values for men and women aged 60–69, at 23.6 ml/kg/min [23]. The number of participants who changed their hypertensive or diuretic medication (commenced, ceased or no change) was not significantly different between the control and LI groups (Table 3).

**Table 2:** Baseline characteristics

Variable	Control (*n* = 68)	LI (*n* = 74)	*p* value
Age (years)	63.5[9.4]	60.5[14.2]	0.23
Male sex, n(%)	41(60.3)	44(59.5)	0.92
African, n(%)	0(0)	1(1.4)	0.34
Diabetes, n(%)	27(42.2)	32(43.8)	0.85
Systolic blood pressure (mmHg)	133[26.5]	130[18]	0.18
Diastolic blood pressure (mmHg)	80[12]	78[10]	0.56
Medications			
ACE inhibitor, n(%)	34(53.1)	34(47.9)	0.54
ARB, n(%)	30(45.5)	44(59.5)	0.10
Thiazide, n(%)	16(25)	13(18.3)	0.35
Spironolactone, n(%)	1(1.4)	3(4.9)	0.89
Loop diuretics, n(%)	14(20.6)	15(21.2)	0.24
Statin, n(%)	41(64.1)	46(64.8)	0.93
Primary cause of renal disease			
Glomerular nephritis, n(%)	2(2.9)	7(9.5)	0.11
Analgesic nephropathy, n(%)	0(0)	2(2.7)	
Renal vascular disease, n(%)	5(7.4)	5(6.8)	0.89
Polycystic kidney disease, n(%)	4(5.9)	4(5.4)	0.90
Reflux nephropathy, n(%)	1(1.5)	1(1.4)	0.95
Pyelonenephritis, n(%)	1(1.5)	1(1.4)	0.95
Calculi, n(%)	0(0)	1(1.4)	
Type 1 diabetes (insulin), n(%)	0(0)	1(1.4)	
Type 2 diabetes (non-insulin), n(%)	3(4.4)	5(6.8)	0.55
Type 2 diabetes (insulin), n(%)	10(14.7)	5(6.8)	0.12
Focal segmental glomerulosclerosis, n(%)	3(4.4)	2(2.7)	0.58
IgA nephropathy, n(%)	4(5.9)	4(5.4)	0.90
Other, n(%)	24(35.3)	21(28.4)	0.38
Unknown, n(%)	5(7.4)	6(8.1)	0.87

*ACE* angiotensin-converting-enzyme, *ARB* angiotensin receptor blocker. Median[IQR] and n(%) is reported

**Table 3:** Change in hypertensive and diuretic medications during the 12-month study period

Medication	Control		LI		*P* value
	Commenced	Ceased	Commenced	Ceased	
ACE inhibitor, n(%)	+ 1(1.5)	−5(7.4)	+ 1(1.4)	−7(9.5)	0.83
ATRB, n(%)	+ 4(5.9)	− 2(2.9)	+6(8.1)	− 6(8.1)	0.30
Thiazide, n(%)	+ 1(1.5)	−2(2.9)	+ 1(1.4)	−3(4.1)	0.90
Spironolactone, n(%)	0	−2(2.9)	0	0	
Loop diuretics, n(%)	+6(8.8)	−3(4.4)	+ 4(5.4)	−3(4.1)	0.76

*ACE* angiotensin-converting-enzyme, *ATRB* angiotensin receptor blocker

### Association between creatinine and lean mass and strength

Table 4 shows correlations between kidney function estimates with fitness and body composition measures in all patients at baseline. Creatinine was significantly associated with lean mass (*r* = 0.32, *p* < 0.01) and grip strength (*r* = 0.39, *p* < 0.001). Cystatin-C however was not correlated with lean mass or grip strength.

**Table 4:** Associations between kidney function estimates with fitness and body composition in all patients at baseline

	Baseline (r value)					
	Creatinine	Cystatin-C	EPIcr	EPIcys	EPIcr-cys	MDRD
VO_2_peak	0.11	−0.04	0.20*	0.16	0.20*	0.18*
VO_2_peak/lean	− 0.15	− 0.10	0.17	0.12	0.15	0.12
Appendicular lean mass	0.32**	0.12	−0.06	0.004	−0.01	− 0.05
Grip strength	0.39**	0.02	0.01	0.11	0.08	0.02

** = *p* <0.01, * = *p* <0.05

### Fitness measures

An additional file demonstrates the baseline and within group changes in all patient characteristics in the LI and Control groups (see Additional file 1). Compared to the Control group, the LI group had significant (*p* < 0.05) improvements in VO_2_peak and METs and a close to significant (*p* = 0.05) increase in 6-min walk time. There were also significant treatment effects with get up and go time, moderate intensity activity, time spent walking and total activity, such that the intervention group demonstrated increases in these variables. There were no significant (*p* > 0.05) between-group differences in appendicular lean mass, grip strength or vigorous intensity exercise. There were also no within group changes in lean mass or vigorous intensity exercise in the LI group.

### Kidney function

The additional file shows no significant within group changes in the LI group for any of the kidney function measures (see Additional file 1). The Control group had a significant increase in cystatin-C over 12 months with subsequent decreases in CKD-EPIcys and CKD-EPIcr-cys. There were no significant between group differences for any of the changes in eGFR measures over the 12 months. A multiple linear regression also identified no association between change in each of the eGFR measures, and change in appendicular lean mass, maximal grip strength or physical activity (Table 5).

**Table 5:** Change in appendicular lean mass, total physical activity time and grip strength on change in eGFR, independent of baseline eGFR using multiple linear regression

	β	*P* value
Delta MDRD (r^2^ = 0.206, *p* = 0.24)		
Delta Appendicular lean mass	−0.15	0.45
Delta maximal grip strength	−0.39	0.06
Delta total physical activity time	0.07	0.75
Baseline MDRD	0.220	0.28
Delta CKD-EPIcr (r^2^ = 0.181, *p* = 0.31)		
Delta Appendicular lean mass	−0.17	0.40
Delta maximal grip strength	−0.37	0.07
Delta total physical activity time	0.07	0.73
Baseline CKD-EPIcr	0.12	0.55
Delta CKD-EPIcys (r^2^ = 0.293, *p* = 0.12)		
Delta Appendicular lean mass	0.07	0.73
Delta maximal grip strength	0.03	0.90
Delta total physical activity time	0.28	0.16
Baseline CKD-EPIcys	−0.43	0.04
Delta CKD-EPIcr-cys (r^2^ = 0.157, *p* = 0.47)		
Delta Appendicular lean mass	−0.10	0.62
Delta maximal grip strength	−0.25	0.26
Delta total physical activity time	0.17	0.45
Baseline CKD-EPIcr-cys	−0.20	0.35

### Agreement of eGFR measures

**Fig. 2:**
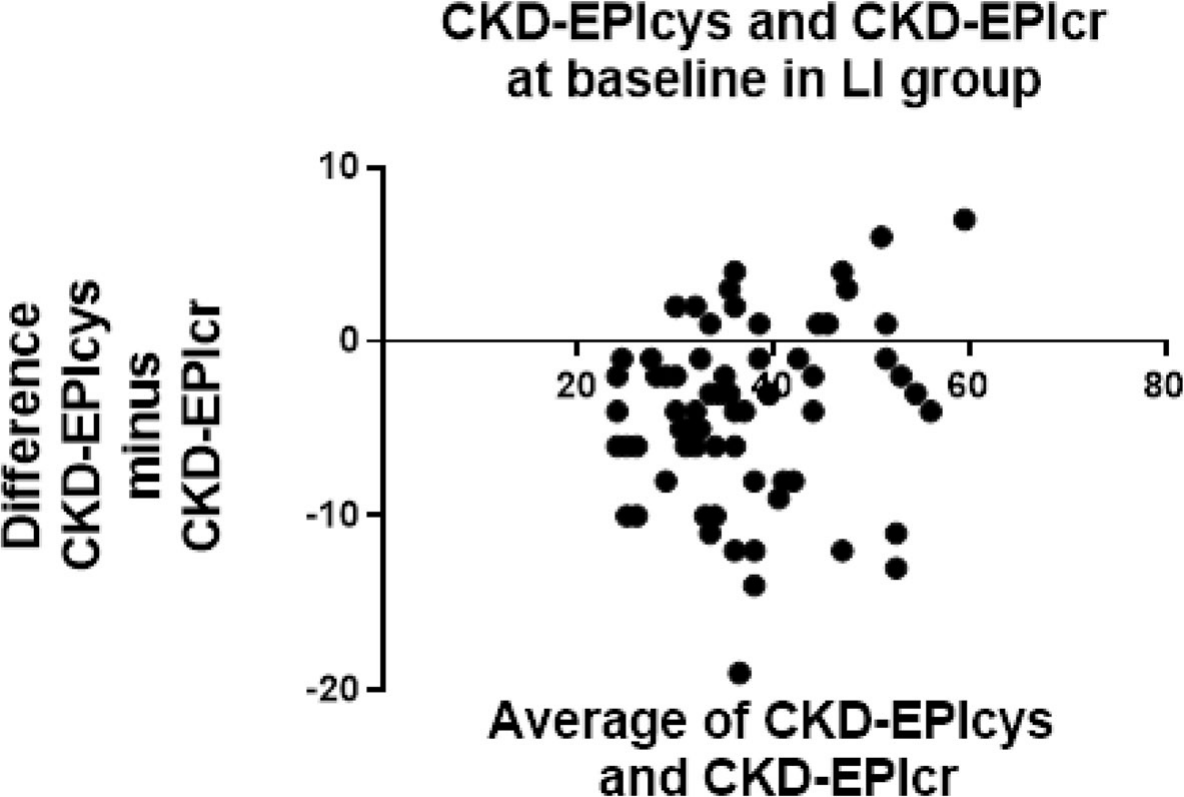
Bland-Altman plots at baseline in LI patients for CKD-EPIcr compared to CKD-EPIcys. CKD-EPIcys minus CKD-EPIcr is divided by the mean of CKD-EPIcys+CKD-EPIcr. CKD-EPIcys is shown to be 7.9 ± 8.6 mL/min/1.73m^2^ less than CKD-EPIcr at baseline

**Fig. 3:**
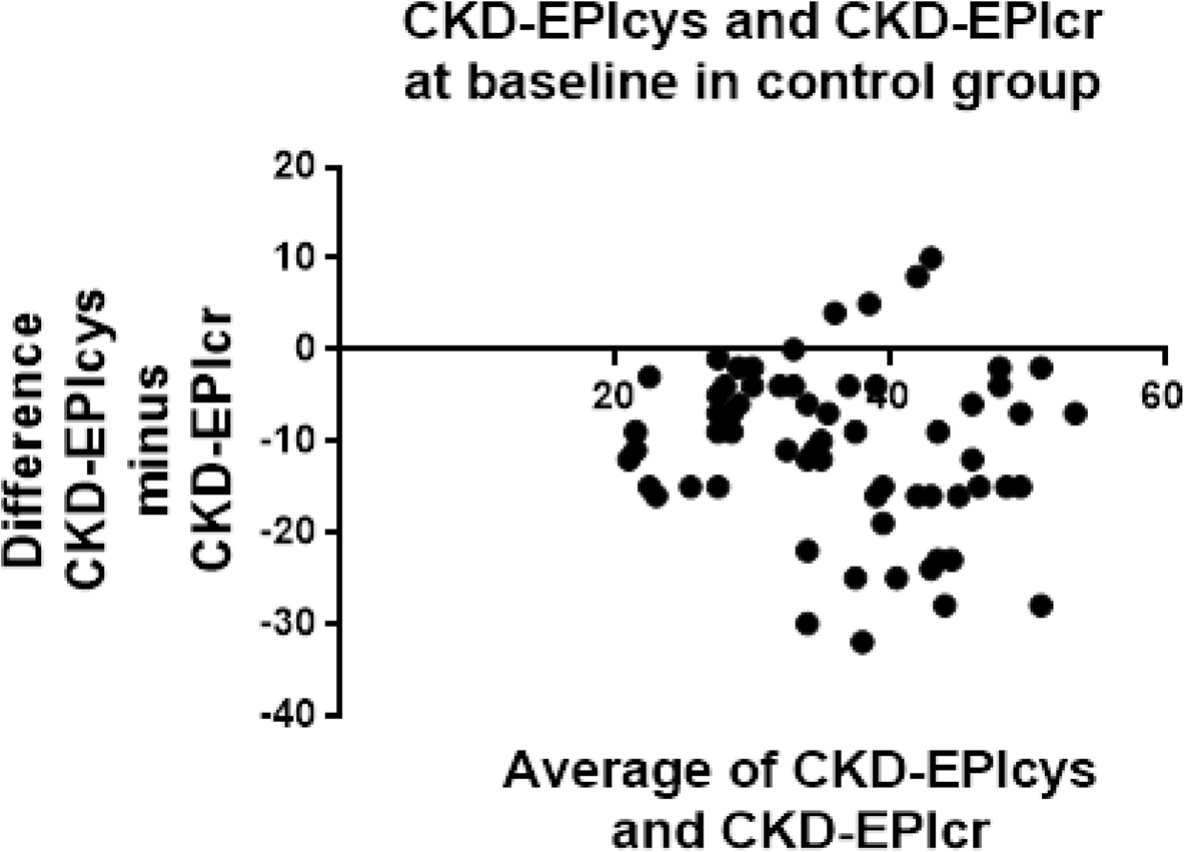
Bland-Altman plots at baseline in the control group for CKD-EPIcr compared to CKD-EPIcys. CKD-EPIcys minus CKD-EPIcr is divided by the mean of CKD-EPIcys+CKD-EPIcr. CKD-EPIcys is shown to be 10.5 ± 9.1 mL/min/1.73m^2^ less than CKD-EPIcr at baseline

**Fig. 4:**
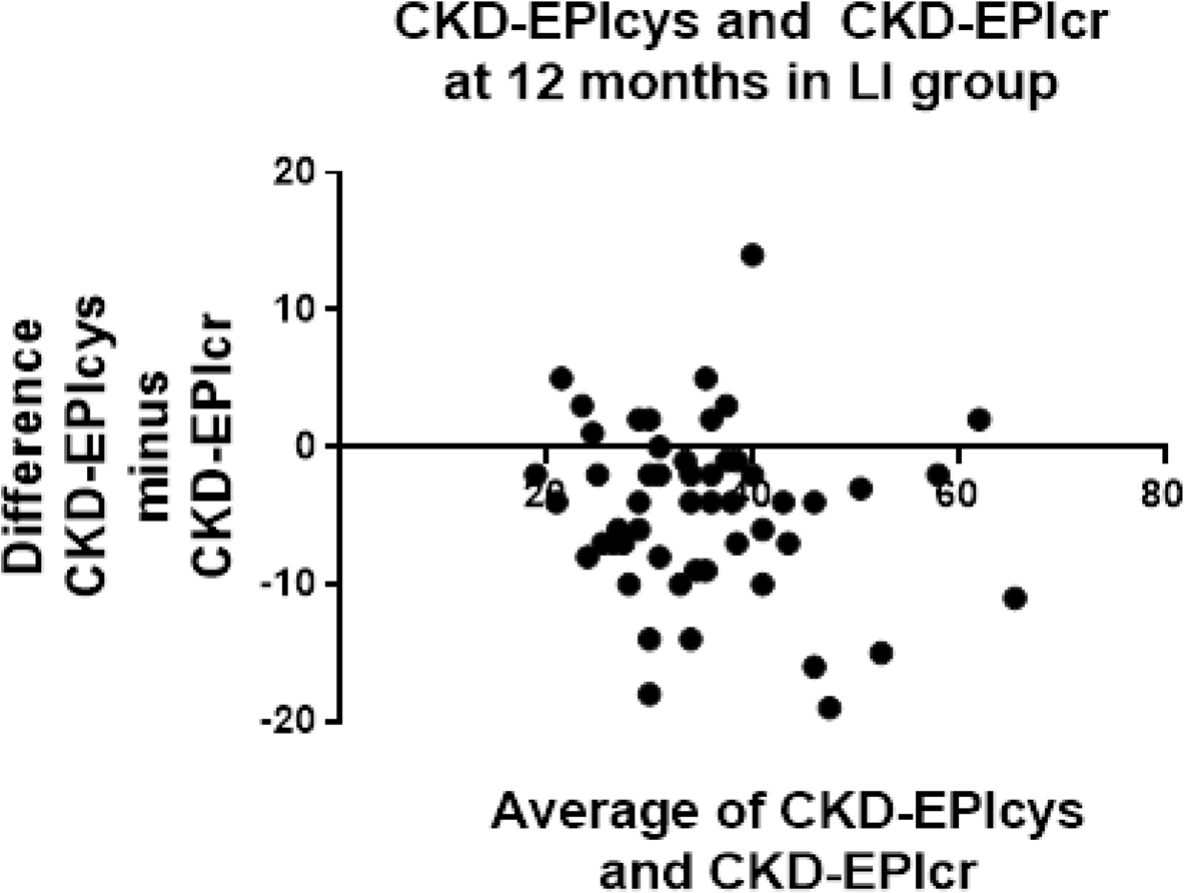
Bland-Altman plots at 12 months in LI patients for CKD-EPIcr compared to CKD-EPIcys. CKD-EPIcys minus CKD-EPIcr is divided by the mean of CKD-EPIcys+CKD-EPIcr. CKD-EPIcys is shown to be 8.4 ± 12.3 mL/min/1.73m^**2**^ less than CKD-EPIcr

**Fig. 5:**
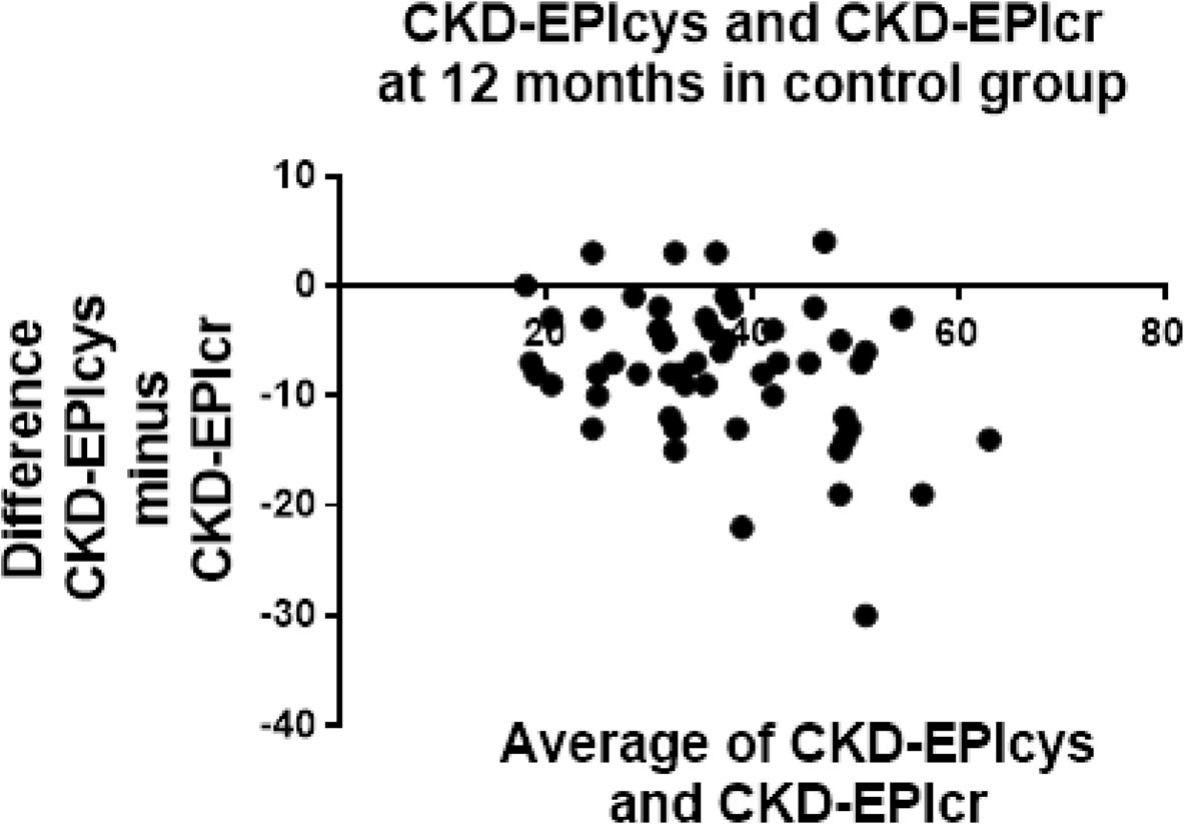
Bland-Altman plots at 12 months in the control group for CKD-EPIcr compared to CKD-EPIcys. CKD-EPIcys minus CKD-EPIcr is divided by the mean of CKD-EPIcys+CKD-EPIcr. CKD-EPIcys is shown to be 13.1 ± 11.8 mL/min/1.73m^**2**^ less than CKD-EPIcr

Tables 6 and 7 shows the agreement between CKD-EPIcys with the other three estimates at baseline in both LI and Control groups. The Bland-Altman analysis identified CKD-EPIcys and CKD-EPIcr-cys to be considerably lower than CKD-EPIcr and MDRDcr for estimating GFR. Figures 2 and 3 indicates CKD-EPIcys records lower average values than CKD-EPIcr at baseline in both LI (eGFR 7.9 below CKD-EPIcr) and Control groups (eGFR 10.5 below CKD-EPIcr). This difference was consistent at 12 months in both LI (eGFR 8.4 below CKD-EPIcr) (Table 8) and Control groups (eGFR 13.1 below CKD-EPIcr) (Table 9). Only CKD-EPIcys and CKD-EPIcr are reported in Figs. 2, 3, 4 and 5, to demonstrate the difference between cystatin-C and creatinine using similar equations (CKD-EPI).

**Table 6:** Bland-Altman agreement of eGFR at baseline in LI patients

	EPIcr	EPIcr-cys	MDRDcr
EPIcys	−7.9 ± 8.6	−3.6 ± 3.7	−6.4 ± 8.4
EPIcr		5.3 ± 5.0	1.5 ± 3.0
EPIcr-cys			− 4.0 ± 5.0

Data is reported as the difference ± SD of the difference

**Table 7:** Bland-Altman agreement of eGFR at baseline in Control patients

	EPIcr	EPIcr-cys	MDRDcr
EPIcys	−10.5 ± 9.1	−3.6 ± 3.7	−9.3 ± 8.4
EPIcr		6.9 ± 5.5	1.4 ± 1.9
EPIcr-cys			−4.0 ± 5.0

Data is reported as the difference ± SD of the difference

**Table 8:** Bland-Altman agreement of eGFR at 12 months in LI patients

	EPIcr	EPIcr-cys	MDRDcr
EPIcys	−8.4 ± 12.3	−2.5 ± 5.5	−6.9 ± 11.2
EPIcr		5.9 ± 6.9	2.2 ± 5.0
EPIcr-cys			−4.3 ± 6.2

Data is reported as the difference ± SD of the difference

**Table 9:** Bland-Altman agreement of eGFR at 12 months in Control patients

	EPIcr	EPIcr-cys	MDRDcr
EPIcys	−13.1 ± 11.8	− 4.5 ± 4.5	−12.0 ± 10.7
EPIcr		8.6 ± 7.4	1.1 ± 2.2
EPIcr-cys			−7.5 ± 6.4

Data is reported as the difference ± SD of the difference

## Discussion

This is the first study to investigate the agreement between cystatin-C and creatinine based eGFR measures before and after a 12-month exercise intervention. The findings from this study indicate that 1) cystatin-C based eGFR estimates were considerably lower than creatinine eGFR estimates; 2) lean mass and grip strength were correlated with creatinine and *not* cystatin-C at baseline; and 3) the agreement between cystatin-C and creatinine eGFR was consistent at baseline and after the LI.

The Bland-Altman analysis showed a considerably lower eGFR measured by CKD-EPIcys than by CKD-EPIcr and MDRDcr. Not surprisingly, the agreement between CKD-EPIcr and MDRDcr was also significant. Furthermore, as expected, the difference between CKD-EPIcr-cys was midway between CKD-EPIcr and MDRDcr, and CKD-EPIcys. An overestimation of eGFR using the MDRDcr equation has previously been reported by Lamb, et al. [24]. CKD-EPIcr is thought to produce less biased estimates of GFR at higher levels of kidney function, however is less accurate when GFR falls below 60 ml/min/1.73m^2^ [25]. On the other hand, cystatin-C is suggested to be a more sensitive marker of GFR than creatinine [26]. This is supported in our study, evident by the lower CKD-EPIcys and CKD-EPIcr-cys identified by the Bland-Altman plots when compared to CKD-EPIcr and MDRDcr. The lower lean mass and strength in the study population [27] may be contributing to the higher CKD-EPIcr and MDRDcr measures compared to cystatin-C. Despite the lower CKD-EPIcys at baseline, it is important to note the agreement between the cystatin-C and creatinine based eGFR measures do not significantly differ after 12 months of exercise training. Due to the routine measurement of CKD-EPIcr and MDRDcr in standard clinical care, these findings support the use of creatinine based eGFR measures in exercising individuals.

The findings from this study identified that lean mass and grip strength were correlated with creatinine and *not* cystatin-C at baseline. This finding is in agreement with evidence that lean mass is significantly related to serum and urinary creatinine but not with cystatin-C, even after adjustment for physical activity levels [11]. From the baseline findings of creatinine correlating with lean mass and grip strength, it would appear that muscle mass and strength would be influencing creatinine based eGFR estimates (CKD-EPIcr and MDRDcr). Although the small sample size limits the ability to detect a significant change in eGFR, the findings indicate that an exercise intervention such as the one reported in this study, does not influence creatinine based eGFR measures.

The equations used to estimate GFR have advanced in precision and accuracy. In 1999 the MDRDcr equation was suggested to be more accurate than the accepted Cockcroft-Gault and creatinine clearance methods [28]. More recently it was proposed that CKD-EPIcr provided a more precise estimate of GFR and categorisation for risk of mortality compared to the MDRDcr study equation [29]. CKD-EPIcr was developed using the same variables as MDRDcr but with different coefficients, which created a moderate improvement in overall accuracy [30]. It has been suggested that standardised serum creatinine assays be used as a first test for assessing eGFR in adults due to its cost-effectiveness, and that Cystatin-C be used as a more precise confirmatory test if a below normal creatinine eGFR is detected [8].

Banfi, et al. [31] identified elevated serum creatinine concentration levels in athletes compared to sedentary controls, proposed to be attributable to higher muscle mass. It is also suggested that the observed eGFR reductions are limited to periods when the athlete is unaccustomed to the training load. This is not unlike a CKD patient commencing an exercise program after an extended sedentary period. However, it is promising to note there was no reduction in eGFR in the intervention group of the current study, despite a large increase in physical activity levels. This is supported by a number of studies who have shown maintenance of kidney function with exercise training [16, 32]. An intervention resulting in significant increases in strength and mass, such as with a specific hypertrophy resistance training program, may potentially provoke a decline in eGFR using creatinine based equations. Indeed, the lack of change in lean mass in the current study limits the ability to make conclusions regarding the effects of exercise induced gains in lean mass on eGFR measures. As such, clinical decisions based on creatinine based eGFR’s of patients undertaking hypertrophy training should be considered with caution. Although the small sample size limits the ability to detect a significant change in eGFR, [25] the findings from this study suggest that primarily aerobic based exercise training with limited resistance exercise, is not enough to elicit changes in eGFR. Future studies should investigate whether specific hypertrophy interventions influence creatinine based eGFR measures. It was not expected that there would be no changes in grip strength and lean mass in the current study. However, in this generalizable CKD cohort the lack of increase in grip strength and lean mass is an important finding and warrants further investigation.

Cystatin-C also has some reported limitations in its use as a kidney function measure, due to its associations with cardiovascular risk factors. After modelling to adjust for measured GFR, Rule, et al. [33] found residual associations of CKD-EPIcys with CKD risk factors, including hypertension, BMI, and c-reactive protein (CRP). This confounding association makes it difficult to establish whether cystatin-C is a true measure of renal function or rather a reflection of cardiovascular disease risk factors [12]. Due to the limitations of both endogenous kidney function measures, it has been suggested that using the combination of creatinine and cystatin-C used in the CKD-EPI equation provides the most accurate assessment of kidney function [8, 33, 34].

This study had some limitations. Physical activity undertaken in the 24-h period preceding testing was not recorded. If the exercise was of high enough intensity it may potentially have resulted in transient increases in serum creatinine. Nevertheless, in the current study testing was completed in the morning after an overnight fast, and participants were asked to refrain from any strenuous exercise the morning of the test. Patients who begin taking, or have an increase in dose of angiotensin converting enzyme inhibitor, angiotensin receptor blocker, famotidine, ranitidine and antibiotics trimethoprim-sulfamethoxazol, H2-blocker cimetidine and cefoxitin may have an increase in serum creatinine [35]. As the dose of medications was not recorded, the impact of these on any potential changes in eGFR creatinine equations is unknown and is therefore a limitation of the study.

A significant limitation which needs to be addressed is the large sample size needed to see a change in eGFR. As suggested by Lamb, et al. [25], 1000 participants are needed to detect differences in accuracy of measurement between MDRDcr and CKD-EPIcys. The authors of this pilot study found in 1000 subjects, the simulations showed an 87% power at the 5% significance level to detect a difference of 5%. The current study did not have the resources to provide longitudinal exercise training on this scale. It is for this reason that the focus of this study was on the agreement between the measures, rather than assessing statistical changes. Future large-scale exercise interventions comparing creatinine and cystatin-C estimates of GFR measures are needed to explicitly address the hypothesis from this study.

## Conclusions

The findings from this study found no difference in the agreement between creatinine and cystatin-C estimates of GFR after a 12-month LI. If only creatinine-based measures are accessible to the treating Physician, the findings from this study suggest it is reasonable to expect creatinine based measures to be a sufficient measure after prescription of a health-enhancing exercise program. Future exercise training studies comparing cystatin-C and creatinine based eGFR measures in specific hypertrophy and high intensity training programs are warranted to confirm the current findings.

## Additional file

Additional file 1: Change in patient characteristics. This table demonstrates the baseline and within group changes in all patient characteristics in the LI and Control groups. (DOCX 15 kb)

## Abbreviations

AA: Active Australia questionnaire
BMI: bBody mass index
CKD: Chronic kidney disease
CKD-EPI: Chronic kidney disease-epidemiology collaboration
Cr-cys: Creatinine-Cystatin-C
Cys: Cystatin-C
DEXA: Dual energy x-ray absorptiometry
GFR: Glomerular filtration rate
IQR: Interquartile range
LANDMARK III: Longitudinal assessment of numerous discrete modifications of atherosclerotic risk kidney disease
LI: Lifestyle intervention
MDRDcr: Modification of diet in renal disease-creatinine
METs: Metabolic equivalent tasks
SD: Standard deviation
