# Supplementary material for: Agreement between cystatin-C and creatinine based eGFR estimates after a 12-month exercise intervention in patients with chronic kidney disease
Source: BMC Nephrol. 2018 Dec 18;19:366. doi: 10.1186/s12882-018-1146-4 (PMC6299617; doi:10.1186/s12882-018-1146-4)
Supplement: Supplementary file 1 — Change in patient characteristics. This table demonstrates the baseline and within group changes in all patient characteristics in the LI and Control groups. (DOCX 15 kb) [file 12882_2018_1146_MOESM1_ESM.docx]

Additional file 1 Change in patient characteristics, exercise parameters and eGFR measurements from baseline to 12 months

| **Variable** | **Control** | | **LI** | | | | **p value** |
| --- | --- | --- | --- | --- | --- | --- | --- |
|  | **Baseline** | **Δ 12m** | **Baseline** | | **Δ 12m** | |  |
| Weight (kg) | 94.9±22.2 | **1.3±4.5*** | 93.9±20.3 | | **-1.8±6.1*^#^** | | **<0.01** |
| Body mass index (kg/m^2^) | 33.3±6.9 | **0.5±1.6*** | 33±6.1 | | **-0.6±2.2*^#^** | | **<0.01** |
| VO_2_peak (ml/kg/min) | 23.2±5.4 | **-0.9±4.2*** | 23.2±7.6 | | **1.7±3.7*^#^** | | **<0.01** |
| Peak METs | 7.5±2.6 | -0.1±1.9 | 7.5±3.7 | | **1.9±1.8*^#^** | | **<0.001** |
| 6 min walk (m) | 483.8±95.9 | 3.0±67.9 | 493.8±106.4 | | **29.5±63.9*** | | **0.05** |
| Grip strength (kg) | 32.8±10.3 | -1.6±6.7 | 35.4±11.8 | | **-2.2±7.2*** | | 0.67 |
| Get up and go (s) | 4.8(1.3) | **0.4(1.1)*** | 5.1(2.0) | | **0.0(1.1)^#^** | | **0.02** |
| Appendicular lean mass (kg) | 23.6±5.7 | 0.0±0.0 | 24.1±5.6 | | 0.0±0.0 | | 0.31 |
| Walking/week (hours) | 0.7(2.3) | 0.0(1.0) | 0.7(2.0) | | **0.5(1.6)*^#^** | | **0.02** |
| Moderate/week (hours) | 0.0(0.0) | 0.0(0.0) | 0.0(0.0) | | **0.2(1.5)*^#^** | | **<0.01** |
| Vigorous/week (hours) | 0.0(0.0) | 0.0(0.0) | 0.0(0.0) | | 0.0(0.3) | | 0.27 |
| Total activity (hours) | 1.2(3.5) | 0.0(2.7) | 1.0(3.0) | | **1.8(3.2)*^#^** | | **<0.01** |
| Total activity (METhours) | 3.8(13.5) | 0.0(11.0) | 3.3(11.8) | | **8(13.7)*^#^** | | **<0.01** |
| **Kidney function measures** |  | | | | | | |
| Creatinine (µmol/L) | 143.8±33.3 | 4.6±26.8 | | 150.3±33.9 | 6.3±29.2 | 0.75 | |
| Cystatin-C (mg/L) | 2.1±0.5 | **0.2±0.4*** | | 2.1±0.5 | 0.1±0.4 | 0.14 | |
| MDRDcr (ml/min/1.73m^2^) | 40.5±8.9 | -0.2±7.5 | | 38.9±8.5 | -1.6±6.7 | 0.29 | |
| CKD-EPIcr (ml/min/1.73m^2^) | 41.9±10.1 | -0.2±8.4 | | 40.7±9.6 | -0.8±8.9 | 0.67 | |
| CKD-EPIcys (ml/min/1.73m^2^) | 30.4±8.9 | **-2.4±6.2*** | | 31.5±10.3 | -0.7±7 | 0.17 | |
| CKD-EPIcr-cys (ml/min/1.73m^2^) | 34.5±8.5 | **-1.5±6.3*** | | 34.6±9.4 | -1.5±5.8 | 0.97 | |
| Protein:creatinine | 38.0(85.5) | -0.5(23.8) | | 38.0(88.8) | -1.0(32.8) | 0.42 | |
| Albumin:creatinine | 14.0(63.5) | 0.0(21.5) | | 14.5(69.4) | 0.0(17.7) | 0.47 | |
| Creatine Kinase | 114.0(124.0) | -12.0(75.0) | | 110.0(91.5) | 0.0(50.0) | 0.22 | |

Delta is calculated by 12 month minus baseline. Mean ± standard deviation is presented for normally distributed delta variables. Median(IQR) is presented for not normally distributed delta variables. P value presented indicates significance of delta values between groups.

*= within group statistical significance, **^#^=** between group statistical significance of change from baseline to 12 months
